# An Under-Recognized Manifestation of Systemic Sclerosis: Macrovascular Peripheral Arterial Disease

**DOI:** 10.7759/cureus.101734

**Published:** 2026-01-17

**Authors:** Meriem Mouharir, Said Kaddouri, Zakaria Chahbi, Hassan Qacif, Mohamed Zyani

**Affiliations:** 1 Department of Internal Medicine, Avicenne Military Hospital / Cadi Ayyad University, Marrakesh, MAR

**Keywords:** case report, ct angiography, intermittent claudication, macrovascular involvement, peripheral arterial disease, systemic sclerosis

## Abstract

Systemic sclerosis (SSc) is a rare autoimmune connective tissue disease characterized by skin and visceral fibrosis and by a well-known microangiopathy leading to Raynaud’s phenomenon, digital ischemia, pulmonary arterial hypertension, and scleroderma renal crisis. By contrast, macrovascular involvement has been less extensively described and may be under-recognized. We report the case of a 66-year-old woman with a 16-year history of SSc. The diagnosis was based on cutaneous sclerosis, Raynaud’s phenomenon complicated by digital necrosis, positive antinuclear antibodies, and interstitial lung disease with pulmonary fibrosis. She was admitted for intermittent claudication of the lower limbs. CT angiography of the lower extremities revealed diffusely small-caliber arteries with long, thread-like stenoses, consistent with macrovascular peripheral arterial involvement in the context of SSc. This case illustrates that SSc may be associated not only with microvascular but also with clinically significant macrovascular disease. Awareness of this complication and the use of vascular imaging in symptomatic patients are essential for early diagnosis and appropriate management.

## Introduction

Systemic sclerosis (SSc) is a chronic autoimmune connective tissue disease characterized by skin and visceral fibrosis and a distinctive microangiopathy [1]. Vascular involvement classically affects the microcirculation and is responsible for Raynaud’s phenomenon, digital ischemia and ulcers, characteristic nailfold capillaroscopic changes, pulmonary arterial hypertension, and scleroderma renal crisis [1-3]. By contrast, macrovascular disease involving medium and large-sized arteries has been less well described and is probably under-recognized in daily practice [2,3]. Several mechanisms have been proposed, including accelerated atherosclerosis, immune-mediated endothelial dysfunction, and structural remodeling secondary to chronic distal microvascular damage [3,4]. The objective of this case report is to describe lower-limb macrovascular peripheral arterial disease in a patient with long-standing SSc and to highlight the importance of considering and actively investigating peripheral arterial disease in this population.

## Case presentation

A 66-year-old woman with a 16-year history of SSc was followed in our internal medicine department. The diagnosis of SSc had been established on the basis of progressive cutaneous sclerosis, Raynaud’s phenomenon complicated by digital necrosis, positive antinuclear antibodies, and interstitial lung disease with radiologic evidence of pulmonary fibrosis. She had been managed with vasodilator therapy and supportive care for Raynaud’s phenomenon and digital ischemia, as well as appropriate treatment for interstitial lung disease.

She was admitted for evaluation of new-onset intermittent claudication of the lower limbs. She reported calf pain induced by walking over a short distance and relieved by rest. There was no history of acute limb ischemia, chest pain, or neurological deficit. The patient had no history of smoking, diabetes mellitus, or dyslipidemia, and body mass index was within the normal range. Fasting plasma glucose and lipid profile were normal. On physical examination, skin thickening of the hands and forearms was noted, with sclerodactyly and residual digital scars. Distal pulses of the lower limbs were diminished. There were no trophic lesions of the feet or toes at the time of examination.

**Figure 1:**
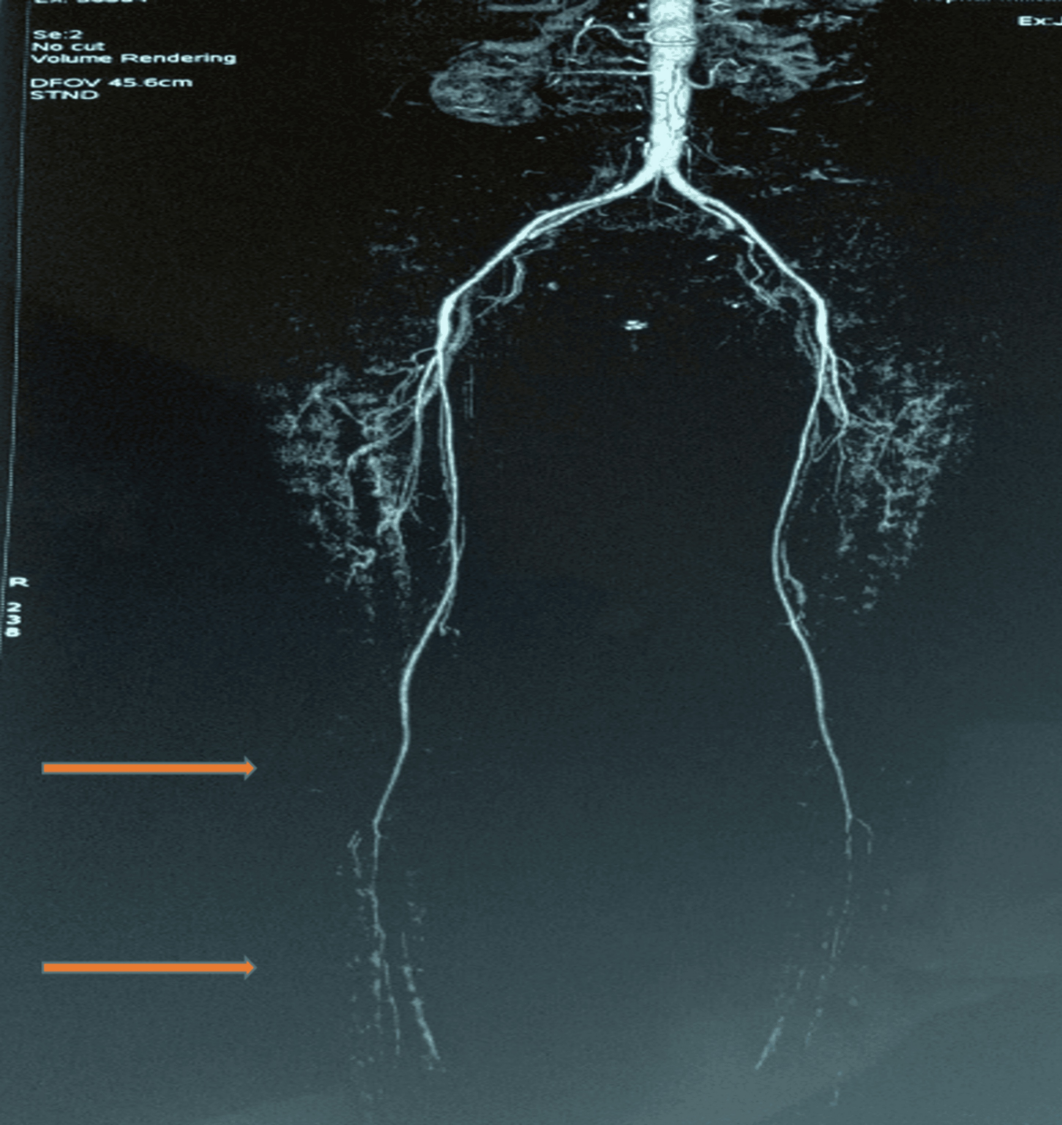
CT angiography of both lower limbs showing diffusely small-caliber arteries with long, filiform (thread-like) stenoses.

Given the clinical suspicion of peripheral arterial disease, CT angiography of the lower extremities was performed. It demonstrated diffusely reduced-caliber arteries with long, concentric, thread-like stenoses involving the main arterial trunks, extending over multiple segments, without focal occlusions, eccentric plaques, or arterial wall calcifications (Figure 1). The lesions showed a smooth, homogeneous narrowing rather than the irregular, segmental morphology typically observed in atherosclerotic disease. This angiographic pattern, combined with the distal predominance of involvement and the coexistence of established microvascular manifestations of SSc, is more consistent with SSc-related macrovascular arteriopathy than with classical atherosclerotic peripheral arterial disease.

Based on the clinical history of long-standing SSc, the presence of microvascular complications (Raynaud’s phenomenon and digital necrosis), and the imaging findings of diffuse distal arterial narrowing, a diagnosis of SSc-related macrovascular peripheral arterial disease was considered. The patient was managed with optimization of vasodilator therapy, statin, antiplatelet treatment, and walking rehabilitation. Clinical follow-up was arranged to monitor symptoms and vascular status.

## Discussion

Microvascular involvement is a well-established feature of SSc and plays a central role in its pathogenesis and clinical expression [3]. Endothelial cell damage, capillary loss, and obliterative microangiopathy contribute to Raynaud’s phenomenon, digital ulceration, pulmonary arterial hypertension, and renal crisis [3,4]. While these microvascular manifestations are well documented and systematically screened for, the presence of macrovascular disease in SSc has been less emphasized.

Macrovascular involvement in SSc can affect medium and large-sized arteries and may present as peripheral arterial disease, particularly of the upper or lower limbs [5]. The pathophysiology of this macroangiopathy remains incompletely understood and is probably multifactorial. Proposed mechanisms include accelerated atherosclerosis, immune-mediated endothelial dysfunction, smooth muscle proliferation and vessel wall remodeling, as well as hemodynamic consequences of chronic distal microvascular obstruction [1,5,6]. Importantly, several studies suggest that macrovascular disease in SSc may occur independently of traditional cardiovascular risk factors, indicating a direct link with the underlying autoimmune vasculopathy rather than “simple” atherosclerosis [4,5].

In our patient, the angiographic findings of diffusely small-caliber arteries with long, filiform (thread-like) stenoses, in the context of long-standing SSc with previous microvascular complications, were highly suggestive of SSc-related macrovascular involvement. The absence of typical focal, plaque-related stenoses favored a non-atherosclerotic process. This observation reinforces the concept that patients with SSc may develop clinically significant peripheral arterial disease, which can manifest as intermittent claudication and carries a risk of progression to critical ischemia if not recognized.

From a practical standpoint, our observation highlights the importance of considering macrovascular disease in SSc patients who report intermittent claudication with exertional limb pain, diminished peripheral pulses, or trophic disorders. Non-invasive vascular imaging such as Doppler ultrasonography, Computed tomography angiography, or magnetic resonance angiography should be performed when there is clinical suspicion of peripheral arterial involvement [1,7]. Early identification allows for timely implementation of appropriate medical management, including optimization of vasodilator therapy, antiplatelet treatment, statins, structured exercise programs, and, in selected cases, endovascular or surgical intervention [3-5].

Recent data indicate that systematic vascular screening reveals a high prevalence of distal arterial involvement, particularly affecting the ulnar arteries in the upper limbs and the posterior tibial arteries in the lower limbs, often in patients without severe traditional cardiovascular risk factors [2,5,6]. Moreover, macrovascular lesions have been associated with trophic disorders, supporting the concept that macrovascular disease represents an intrinsic manifestation of SSc-related vasculopathy [2,4]. These findings underline the need for increased awareness and systematic evaluation of macrovascular involvement in SSc, especially in symptomatic patients, to improve outcomes and prevent severe ischemic complications.

## Conclusions

SSc is classically regarded as a microvascular disease, but macrovascular involvement can also occur and may lead to symptomatic peripheral arterial disease. Our case illustrates this complication. Clinicians should maintain a high index of suspicion for macrovascular disease in SSc, particularly in patients with new-onset limb claudication or diminished peripheral pulses. Vascular imaging plays a key role in diagnosis, and early recognition is crucial to optimize management and prevent progression to critical limb ischemia.
